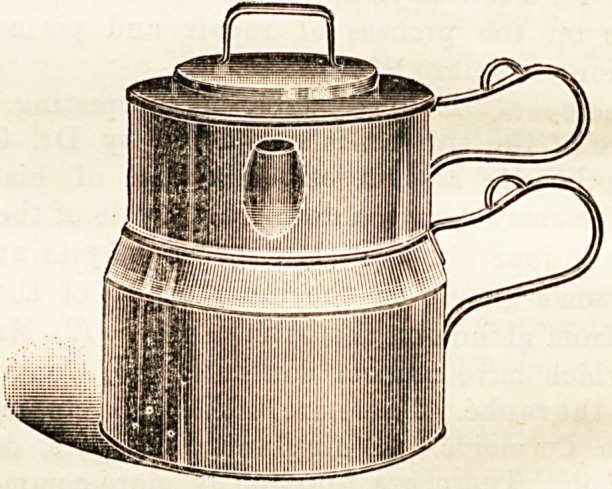# New Appliances and Things Medical

**Published:** 1898-08-27

**Authors:** 


### NEW APPLIANCES AND THINGS MEDICAL.

[We shall be glad to receive, at our Office, 28 & 29, Southampton Street, Strand, London, W.C., from the manufacturers, specimens of all new preparations and appliances which may be brought out from time to time.]

### AYMARD'S PATENT MILK STERILISER COMPANY.

(St. Mathew's Works, Ipswich.)

Since our favourable report upon these sterilisers in our issue of April 18th, 1896, it is pleasing to note that distinct advances have been made, and, as we pointed out at the time, the moderately small sized ones have been mostly in demand for infant and invalid feeding. Heating to a high temperature, and especially prolonged heating, is detrimental to milk. This is avoided in the Aymard steriliser, by which the milk is heated only to the temperature which is necessary to destroy injurious organisms, and is not maintained at that temperature any longer than is required for the purpose. Aymard's sterilisers have been adopted by Her Majesty's War Office, &c., and by various hospitals and kindred institutions. The apparatus is simple, portable, and is easily cleaned—a matter of no small importance.

## Figures and Tables

**Figure f1:**